# Beyond the Bench: Mapping the Air in Public Schools

**DOI:** 10.1289/ehp.113-a308

**Published:** 2005-05

**Authors:** Tanya Tillett

In the home, parents are the primary guardians of a child’s environmental health. However, once the child’s environment moves out of the home, identifying sources of protection can become difficult. The Community Outreach and Education Program (COEP) of Duke University’s NIEHS Environmental Health Sciences Research Center in Durham, North Carolina, has pledged to give children’s environmental health the attention it deserves and is proactively implementing projects to create preventive environmental interventions that protect children before they become sick.

One of the main questions the Duke COEP seeks to answer in its outreach activities is whether children are being exposed to environmental factors in school that contribute to asthma incidence. The Duke COEP staff have used geographic information system (GIS) technology in a number of applications to address this question, including a May 2004 assessment of the indoor air environment at nearby New Hope Elementary School.

For years, students and staff in the Orange County school system (of which New Hope Elementary is a part) had complained of respiratory problems as well as general discomfort with fluctuating indoor temperatures and a sense that the air in the school buildings was unhealthy. COEP staff were asked to evaluate one sample school in the system and offer recommendations. They selected New Hope Elementary for study, then went into the school and conducted an evaluation of every classroom.

The problems they observed included a high relative indoor humidity of around 75–78% (the ideal humidity is 35–40% when the outdoor temperature is 20°F or higher) and imbalances in the heating and cooling system (they observed temperature differences of as much as 5–10°F in separate classrooms in some instances). They also noticed problems exacerbated by failing insulation, such as mold spore development and air flow variations—some rooms would have a distinct breeze, while others had still, damp air.

Once they had gathered their data, the COEP staff created a GIS compilation to display the data and analyze the results. The advantage of using GIS technology to interpret information is that it visually integrates previously unlinked sets of information, facilitating the detection of relationships among them. In the case of New Hope Elementary, the GIS map let concerned parents, teachers, school administrators, and education policy makers see a visual representation of the spatial distribution of airflow irregularities and mold spores throughout the school.

Based on information gathered from the GIS map, the school administrators replaced carpeting throughout the school with tile floors and thoroughly cleaned the entire duct system, which significantly improved ventilation in the classrooms. “What’s exciting is that we were able to provide information, education, and outreach directly to the faculty and school governance council,” says Marie Lynn Miranda, director of the Duke COEP.

Another benefit of the study is the unique learning opportunity it will afford the New Hope Elementary students. All the data from the project will be turned over to the school’s fifth-grade science teachers for development into classroom lesson projects. COEP workers also plan to use the New Hope Elementary study as a model for future initiatives in childhood asthma prevention.

## Figures and Tables

**Figure:**
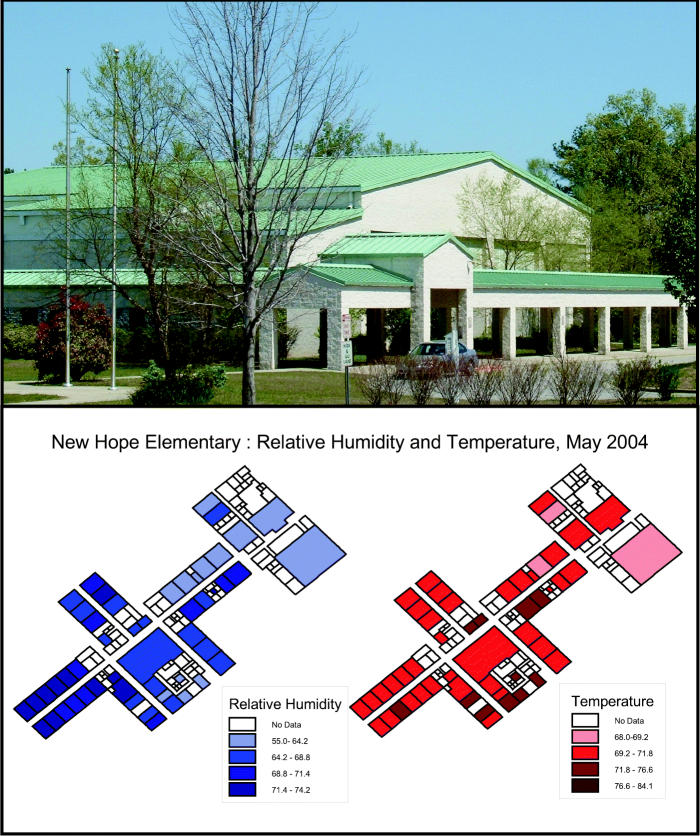
**Mighty map.** A GIS map of Hope Valley Elementary School allowed staff, parents, and administrators to see where there were unhealthful fluctuations in humidity (blue) and temperature (red). With this information in hand, the school was able to take appropriate corrective action.

